# 

**Published:** 1904-09

**Authors:** 


Sixtieth Year.

VOL. LX.
WHOLE NUMBER,
DCLXXXXIV

SEPTEMBER, 1904.

New Series.
VOL. XLIV
No. 2

### BUFFALO MEDICAL JOURNAL

INDEXED
[illegible] G. O.

Established 1845 by AUSTIN FLINT, M. D.

EDITOR:
WILLIAM WARREN POTTER, M. D.

ASSISTANT EDITORS:
WILLIAM C. KRAUSS, M. D. NELSON W. WILSON, M. D.

ASSOCIATE EDITORS:
JAMES WRIGHT PUTNAM, M. D. ERNEST WENDE, M. D.
JOHN PARMENTER, M. D. JOHN A. MILLER, PH. D.
HARVEY R. GAYLORD, M. D. MAUD J. FRYE, M. D.

For terms and other information relating to the Journal, see page vi.

